# Insecurity, distress and mental health: experimental and randomized controlled trials of a psychosocial intervention for youth affected by the Syrian crisis

**DOI:** 10.1111/jcpp.12832

**Published:** 2017-10-02

**Authors:** Catherine Panter‐Brick, Rana Dajani, Mark Eggerman, Sabrina Hermosilla, Amelia Sancilio, Alastair Ager

**Affiliations:** ^1^ Department of Anthropology and Jackson Institute for Global Affairs Yale University New Haven CT USA; ^2^ Department of Biology and Biotechnology Hashemite University Zarqa Jordan; ^3^ MacMillan Center for International and Area Studies Yale University New Haven CT USA; ^4^ Columbia University Medical Center Columbia University New York NY USA; ^5^ Department of Anthropology Yale University New Haven CT USA; ^6^ Institute for Global Health and Development Queen Margaret University Edinburgh UK

**Keywords:** Mental health and psychosocial support, evaluation, forced displacement, refugees, war, implementation science

## Abstract

**Background:**

Strengthening the evidence base for humanitarian interventions that provide psychosocial support to war‐affected youth is a key priority. We tested the impacts of an 8‐week programme of structured activities informed by a profound stress attunement (PSA) framework (*Advancing Adolescents*), delivered in group‐format to 12–18 year‐olds in communities heavily affected by the Syrian crisis. We included both Syrian refugee and Jordanian youth.

**Methods:**

We followed an experimental design, comparing treatment youth and wait‐list controls over two programme implementation cycles, randomizing to study arm in cycle 2 (ClinicalTrials.gov ID: NCT03012451). We measured insecurity, distress, mental health difficulties, prosocial behaviour and post‐traumatic stress symptoms at three time‐points: baseline (*n* = 817 youth; 55% Syrian, 43% female), postintervention (*n* = 463; 54% Syrian, 47% female), and follow‐up (*n* = 212, 58% Syrian, 43% female). Regression models assessed: prospective intervention impacts, adjusting for baseline scores, trauma exposure, age, and gender; differential impacts across levels of trauma exposure and activity‐based modality; and sustained recovery 1 year later. We analysed cycle‐specific and cycle‐pooled data for youth exclusively engaged in *Advancing Adolescents* and for the intent‐to‐treat sample.

**Results:**

We found medium to small effect sizes for all psychosocial outcomes, namely Human Insecurity (*β* = −7.04 (95% CI: −10.90, −3.17), Cohen's *d* = −0.4), Human Distress (*β* = −5.78 (−9.02, −2.54), *d* = −0.3), and Perceived Stress (*β* = −1.92 (−3.05, −0.79), *d* = −0.3); and two secondary mental health outcomes (AYMH:* β* = −3.35 (−4.68, −2.02), *d* = −0.4; SDQ:* β* = −1.46 (−2.42, −0.50), *d* = −0.2). We found no programme impacts for prosocial behaviour or post‐traumatic stress reactions. Beneficial impacts were stronger for youth with exposure to four trauma events or more. While symptoms alleviated for both intervention and control groups over time, there were sustained effects of the intervention on Human Insecurity.

**Conclusions:**

Findings strengthen the evidence base for mental health and psychosocial programming for a generation affected by conflict and forced displacement. We discuss implications for programme implementation and evaluation research.

## Abstract 2

Read the Commentary on this article at doi: 10.1111/jcpp.12870

## Introduction

Protracted conflict represents a major threat to population health globally. Adolescents are especially vulnerable to poor mental health and the disruption of social and developmental trajectories, in ways that affect human wellbeing and the economic development of nations (Viner et al., 2012). Both scientifically and strategically, concrete steps to advance adolescent health have far‐reaching consequences (Resnick, Catalano, Sawyer, Viner, & Patton, 2012), especially in refugee and war‐affected settings where child and adolescent mental health is a key policy, research and humanitarian concern (Betancourt, Meyers‐Ohki, Charrow, & Tol, 2013b; Fazel, Reed, Panter‐Brick, & Stein, 2011; Hassan, Ventevogel, Jefee‐Bahloul, Barkil‐Oteo, & Kirmayer, 2016; Reed, Fazel, Jones, Panter‐Brick, & Stein, 2011; Sami et al., 2014; W. A. Tol, Song, & Jordans, 2013).

Nowhere has this been demonstrated more vividly than in the Syrian crisis, now in its sixth year. As of September 2017, over 5.22 million people have been forced to leave Syria, making this the largest refugee crisis of our time; over 650,000 Syrians have taken refuge in Jordan, half of whom are under 18 years of age (UNHCR, 2017). Syrian refugee youth face the demands of adjustment to living in host communities following prolonged exposure to the toxic stress of insecurity, fear and loss (Save The Children, 2017). Host‐community youth are also deeply affected by the Syrian crisis, given increased pressures on neighbouring countries to accommodate the influx of urban refugees. In Jordan, the large majority (79%) of Syrian refugees live outside refugee camps (UNHCR, 2017).

Interventions that provide mental health and psychosocial support (MHPSS) to war‐affected communities serve a humanitarian imperative to relieve suffering. They may be protective in decreasing psychological problems, or promotive in fostering positive psychological and social outcomes (Tol et al., 2013). They include specialized, clinical mental health services and focused psychosocial support (Bangpan, Dickson, Felix, & Chiumento, 2017; Tol et al., 2011). The evidence base that guides treatment and prevention efforts, however, remains severely limited (Dua et al., 2011; Ehntholt & Yule, 2006). Rigorous studies are skewed towards a focus on post‐traumatic stress disorder (PTSD) and offer mixed findings, especially for age and gender (Jordans, Tol, Komproe, & de Jong, 2009). In‐depth reviews conclude that mental health and psychosocial support (MHPSS) programmes have seldom been robustly evaluated in humanitarian settings (Blanchet et al., 2015; Tol et al., 2011), suggesting an urgent need to strengthen the evidence base (Ager et al., 2014). Several randomized trials in conflict‐affected settings do suggest that brief, structured interventions – potential first‐line treatments for depression, anxiety and post‐traumatic stress disorder – can improve outcomes where carefully implemented (Betancourt et al., 2014; Jordans et al., 2010; Rahman et al., 2016). A critical research gap exists with respect to the impacts of the most frequently implemented psychosocial interventions, namely those aimed at promoting psychosocial wellbeing in children and adolescents, usually delivered in group format for a period of 1–3 months (Bangpan et al., 2017; Tol et al., 2011). Indeed, few programme evaluations have featured explicit comparisons of youth accessing versus not accessing the interventions.

In response to specific calls to strengthen the evidence base for health interventions in humanitarian settings (Blanchet et al., 2015) and to address the mental health needs of Syrian refugees (Quosh, Eloul, & Ajlani, 2013), we partnered with Mercy Corps, a global humanitarian and development agency, to evaluate a psychosocial intervention targeted at a generation of children affected by the Syria crisis. Recognizing that adolescence is a key time for protecting the next generation and building its future, Mercy Corps began implementing its *Advancing Adolescents* programme in 2014 with community‐based partner organizations in Jordan, Lebanon, Iraq, Syria and Turkey (Mercy Corps, 2014a, 2014b). These interventions were funded as part of the *No Lost Generation* initiative, launched in 2013 to address the devastating impacts of the Syria and Iraq crises (No Lost Generation, 2016). The campaign, led jointly by UNICEF, Mercy Corps, Save the Children and World Vision, has the potential to reach 400,000 adolescents in crisis settings in the Middle East (http://www.nolostgeneration.org).

*Advancing Adolescents* [Arabic: *Nubader*] features structured, group‐based activities that draw upon a profound stress attunement (PSA) framework to promote capacities for the mediation of extreme and prolonged stress (Macphail, Niconchuk, & El‐wer, 2017). Mercy Corps adopted the PSA platform to enhance young people's socioemotional development and mental health, support educational and skills‐building training, help with decision‐making, develop empathy and resilience, and build strong ties in the community (Kurtz, 2016). Such programming may mitigate the impact of toxic stress on human development (Shonkoff et al., 2012). Indeed, the lay coaches who deliver the intervention are trained by Mercy Corps to understand how stress physiology affects brain function and how to facilitate experiential learning (Table 1). Programme sessions are made available to both Syrian refugee and host‐community youth, separately for male and female, within urban areas, in safe and familiar community locations (Mercy Corps, 2014a). By 2016 in northern Jordan, Mercy Corps had trained 295 community partners and volunteers on stress attunement, in partnership with community‐based organizations and Jordan's Ministries of Culture, Education and Planning (Mercy Corps, 2016).

**Table 1:** The Advancing Adolescents programme

*Focus*: The *Advancing Adolescents (Arabic: Nubader)* programme is a structured, 8‐week psychosocial intervention for adolescents in humanitarian crises, based on profound stress attunement processes. It features three elements that are widely viewed as important to support youth adjustment in contexts of complex emergencies: (a) safety: establishment of a ‘safe space’ within the community as a base for activities and site of protection, (b) support: facilitation of social support and self‐expression and (c) structured, group‐based activities.
*Beneficiaries*: Refugee and host‐community youth (12–18 years old). Eligibility is based on vulnerability and need, determined by Mercy Corps staff during screening interviews to assess age, self‐reported mental health difficulties and poor access to local services. Siblings are included when families prefer brothers and sisters to travel and participate together.
*Sessions and structured activities*: Sessions are open to gender‐differentiated groups of 8–15 youth, where possible matched for age. A total of sixteen sessions are run, usually two per week, on weekdays or weekends. Sessions start on a rolling basis, once urban centres have obtained governmental authorization, adult coaches have completed training, and adolescents have signed up for specific modalities. Participants are given a choice of programme modalities, which range from fitness activities (nature walks, football), arts & crafts (sewing, graphic design, photography, singing & folklore, drama, theatre sessions), to vocational skills (hairdressing, beautician training) and technical skills (computer repairs, English language, first aid). To build strong community engagement, programme participants also design Community Development Project Plans that link them to community leaders and build social capital.
*Profound Stress Attunement (PSA) approach*: The profound stress attunement approach is a community‐based, nonclinical programme of psychosocial care to meet the psychosocial needs of at‐risk children and improve social interactions with participatory approaches. It focuses on the practice of attunement, for developing safe emotional spaces, managing stressors and establishing healthy relationships. Attunement principles are based on the eight guidelines of the International Child Development Programme (Hundeide, 2013), whereby emotional interactions focus on showing positive feelings of love and affection, responding to the child's initiatives, establishing verbal and nonverbal emotional dialogue, and praising and showing approval, while mediational interactions lead to focusing together on activities and discussions, describing emotions and challenges, stimulating goal formation, memory retention, or creative expression, and practicing positive regulations such as goal planning and behavioural accountability (Macphail et al., 2017). This approach has been used in Southeast Asia, West Africa and the Middle East in programming for child soldiers and other war‐affected child and adolescent populations.
*Practice elements*: As coded in meta‐reviews (Brown et al., 2017), sessions are built for psychoeducation, insight‐building, cognitive strategies, relationship/rapport building, safe place, networking support, talent/skill building, communication skills, motivational enhancement and strategies for maintenance. In addition, sessions include a mix of refugee and nonrefugee youth, in order to promote coexistence and empathy for one another.
*Key innovation*: The Profound Stress Attunement framework draws on neuroscience to communicate understanding of the impacts of long‐term stressors on human emotions and everyday behaviours. Participants are taught to recognize the reactions of the ‘emotional brain’ in response to experiences of profound stress, in order to manage impulse control, assess risk, approach the future with effective skills and strategies, rebuild empathy for self and compassion for others in the community (Mercy Corps 2016).
*Coaches and training programme*: Male and female coaches are lay volunteers (21–60 year‐old) from the local area. They complete a 16‐day training in program delivery, to work as instructors, facilitators, mentors and animators. The *Coaches Foundation Training Programme* focuses on emotional and behavioural regulation (‘Hearts and Heads’) and experiential learning (‘Creative Facilitation’). The manual incorporates the following components: practicing healthy communication; defining profound stress, its impact on the human brain, and principles of attunement; developing gender equity and adolescent protection; building psychosocial resilience; and enhancing skills of creative facilitation for effective technical training. The coaches develop technical training ‘session plans’ to lead structured activities, with guidance from Mercy Corps.
*Quality management and supervision*: Training, implementation, and assessments (e.g. session plans, delivery of technical skills, goal‐setting for youth in development plans, attendance) are undertaken by the Mercy Corps monitoring and evaluation team. Training guidelines reinforce an understanding of key objectives, quality assurance and quality improvement. A lay coordinator monitors and supports the project plans during their development and implementation. Weekly meetings are scheduled to review progress, share experiences and address issues arising. Refresher training courses are offered to lay coaches before each new cycle of implementation. Coordinators and coaches who identify cases of concern use referral pathways modelled after UNHCR.
*Location*: Youth centres, designed as ‘Adolescent Friendly Spaces’ in partnership with local community‐based organizations engaged in building civic society or development training, open 9 am to 9 pm. In northern Jordan, the programme was implemented in the urban centres of Irbid, Jarash, Mafraq, Ajloun and Zarqa governorates, with the support of community‐based organizations and Jordan's Ministry of Culture, Ministry of Education, and Ministry of Planning (Mercy Corps, 2016).

Given the weak evidence base for skills and activity‐based programming of this form and scale (Ager et al., 2014; Blanchet et al., 2015; Tol et al., 2011), we featured three design elements to enhance rigour of programme evaluation. First, we adopted an experimental design, to assess impacts beyond the changes observed in local comparison groups (controls). Second, we featured operational independence between teams responsible for research evaluation and programme implementation, to minimize potential desirability bias in participant responses. Third, we included a broad range of psychosocial, physiological and cognitive outcomes; we report here on psychosocial outcomes. Specific objectives were to test whether outcome measures differed for treatment versus controls, taking into account age, gender, lifetime trauma exposure and programme implementation cycle.

Our *first research question (does the intervention work?)* assessed programme effectiveness in alleviating insecurity and stress, as well as improving mental health difficulties and prosocial behaviour. Our main hypothesis was that the specific orientation to profound stress attunement, taken in delivering *Advancing Adolescents*, would alleviate, in youth, a sense of insecurity and distress. Further, it would improve mental health difficulties and enhance prosocial capacities in refugee and host communities. Our *second question (for whom does the intervention work?)* concerned differential treatment effects, with respect to trauma exposures, gender, age and programme modality. Our hypothesis was that differential trauma exposures for war‐affected youth in refugee and host communities would have discernable impacts on programme responsiveness. Our *third question (are intervention effects sustained?)* examined impacts over the longer term with follow‐up data collected around 1 year after programme completion. In the absence of additional programming, we expected potential impacts to attenuate for youth exposed to the intervention, affecting recovery over time.

## Methods

### Intervention and setting

The intervention was run by Mercy Corps, whose staff screened and enrolled youth residing in Jordanian urban centres into five cycles of programme delivery (2014–2016). Consisting of sixteen sessions (over 8 weeks), the intervention explicitly integrated Syrian refugees and Jordanian hosts in gender‐differentiated groups of ten to fifteen youth per group, age‐matched where possible (Table 1). The sessions were run by adult lay facilitators from the local community, trained by Mercy Corps to enhance safety and psychosocial support while imparting life skills for vulnerable children and adolescents. Eligibility of beneficiaries (refugees and host‐community youth, 12–18 years old) was determined by Mercy Corps staff through screening for children's mental health difficulties and poor access to basic services. Youth chose from a range of programme modalities that included fitness, arts/crafts, vocational skills and technical skills. Transport for participants by bus was provided, where needed, to the community centres hosting registration, training and activities. The programme was implemented on a rolling basis, when official authorization, coach training and modality enrolments were complete.

### Study design

**Figure 1:**
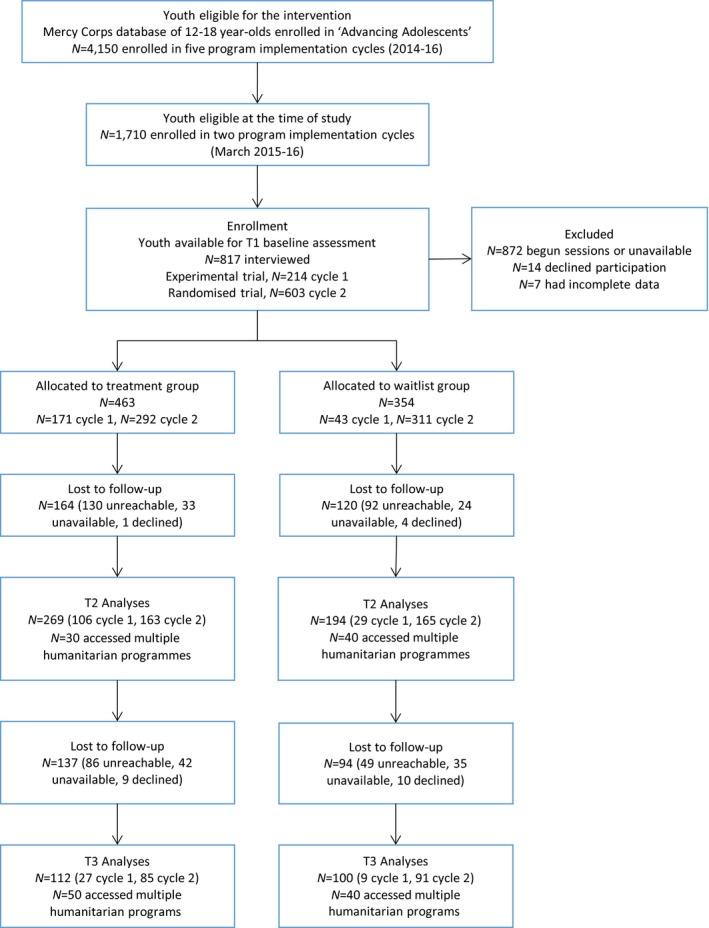
Study design and sample frame [Colour figure can be viewed at http://wileyonlinelibrary.com]

In design and sample size, we followed guidelines to detect 0.3 effect sizes for the evaluation of psychosocial and behavioural therapies; such effect sizes are potentially achievable and clinically significant for youth in humanitarian settings (Ager, Ager, Stavrou, & Boothby, 2011; Betancourt et al., 2014). The research study (March 2015–February 2016) coincided with two programme implementation cycles conducted by Mercy Corps in spring and winter, each implemented in four sites. Using Mercy Corps data for our sampling frame (Figure 1), we recruited adolescents who had enrolled at the time of study and were available for baseline assessment (*n* = 817). This sample represented 20% of the 4,150 young people enrolled by Mercy Corps over five implementation cycles in 2014–16, and 48% of the 1,710 youth enrolled over the two cycles captured by the study. It excluded youth (*n* = 872) who had begun sessions or were otherwise unavailable for participation. There were 14 cases of parent refusals and seven cases with incomplete data, including two cases with mental and physical difficulties preventing communication. Study cycle 1 (March–June 2015) took place in the urban centres of Irbid, Jarash, Mafraq and Ajloun. One author (RD) was responsible for allocation to treatment and control groups: youth who could complete sessions before the fasting month of Ramadan (mid‐June) were assigned to the treatment group, while those unable to participate during this period due to unavailability of trained coaches were wait‐listed as controls. This represented a quasi‐experimental trial: we could not ethically assign participants to treatment/control study arms on the basis of a coin‐toss randomization, since Mercy Corps was not guaranteed funding renewal and could not promise future programming in the area. Study cycle 2 (September 2015–February 2016) was undertaken after donors had renewed funding, for activities in Irbid, Jarash, Mafraq and Zarqa urban centres. Here, we adopted a fully randomized design, with controls explicitly wait‐listed for the next available cycle of programme iteration. Informed consent was given prior to the randomization process, with both participants and fieldworkers blinded to randomization outcome. Families consented to a coin‐toss allocation (ratio 1:1) of lollipop colours to study arms, with each youth selecting one of two coloured lollipops from an opaque cloth bag. Once baseline assessments were complete, one author (RD) completed the coin toss, informing families of an immediate or delayed programme start‐date. This process was completed independently at each site, for each program modality, to accommodate different start‐times of Mercy Corps activities. We re‐interviewed treatment/control youth after 10 weeks, for postintervention data (T2). We scheduled the follow‐up (T3, July–August 2016) between seven and fourteen months (with an average of 11 months) thereafter, scheduling assessments after the period of school exams and avoiding the fasting month of Ramadan.

The study received formal approval from the Prime Minister's Office of Jordan and ethics approval by Yale University, and was registered at ClinicalTrials.gov (https://clinicaltrials.gov/ct2/show/NCT03012451). Written informed consent was obtained from community leaders, parents and adolescents. The evaluation team, recruited and trained independently from Mercy Corps, included Syrian/Jordanian research managers, field supervisors and male/female interviewers. The same personnel undertook all surveys with adolescents, in Arabic; interviews were held in private, concluding with small gifts of toiletries. The sponsor (Elrha's Research for Health in Humanitarian Crises R2HC Programme), funded equally by the Wellcome Trust and the UK Government, had no role in the design or conduct of data collection or analysis.

### Outcomes measures

We measured three dimensions of psychological burden: human insecurity, relevant to contextual experiences of fear and safety; distress, relevant to psychosocial states of frustration and humiliation; and mental health difficulties, relevant to clinical concerns for treatment and prevention. Given the intervention rationale of profound stress attunement, primary outcomes were predefined as the reduction of psychosocial distress and insecurity, with mental health difficulties and prosocial behaviour as secondary outcomes related to potential enhancement of emotional and behavioural self‐regulation. We also included a measure of post‐traumatic stress reactions, due to its salience in war‐related research, as an outcome untargeted by the intervention. We used regionally/internationally validated scales, chosen on the basis of their simplicity, cultural relevance, psychometric properties and usage in conflict areas. All instruments were reviewed by a panel of Syrian/Jordanian scholars and Western academics experienced in mental health research in Middle‐Eastern settings, and piloted with Syrian/Jordanian adolescents.

To assess insecurity and stress, we implemented the *Human Insecurity* (HI) and *Human Distress* (HD) scales, developed and validated in the West Bank with Palestinian populations (Hamayel, Ghandour, Abu Rmeileh, & Giacaman, 2014; Ziadni et al., 2011). The HI (10 items) and HD (12 items) are 5‐point Likert scales with scores expressed on a scale of 0–100. The HI covers issues such as worries regarding inability to obtain daily life necessities, losing a source of income, fears about the future and family safety. For comparison with other studies, high levels of insecurity were calculated as response scores corresponding to always/most of the time (Barber et al., 2016). The HD asks the extent to which youth feel unable to control important things in life, and feel frustrated, incapacitated, humiliated or unable to perform daily activities. To corroborate this measure, we implemented the *Perceived Stress Scale* (PSS; 14 items, 5‐point Likert scale), assessing past‐month feelings of being upset, nervous, angered, lacking control or inability to cope; while PSS was developed in Western contexts, the Arabic‐language PSS was validated in Jordan (Almadi, Cathers, Mansour, & Chow, 2012).

To assess mental health difficulties, we used two scales: the *Arab Youth Mental Health* (AYMH) and the total difficulties subscale of the *Strengths and Difficulties Questionnaire* (SDQ). The former (21‐item, 3‐point scale) was specifically designed for the region and validated as a useful screening tool for depression and anxiety in Lebanese children (Mahfoud et al., 2011). The latter (20 items, 3‐point scale) provides balanced coverage of emotional and behavioural mental health difficulties (Goodman & Goodman, 2009); the youth self‐report version has been validated in Arabic (Almaqrami & Shuwail, 2004; Thabet, Stretch, & Vostanis, 2000), and used with Syrian refugees (Giordano et al., 2014) and other war‐affected populations. Moreover, the Arabic version of the SDQ has been validated for use in epidemiological and clinical studies: it has value for screening at a population level and for predicting psychiatric diagnoses (Alyahri & Goodman, 2006). We used the SDQ prosocial subscale (5 items) to assess functional behaviour. Lastly, we implemented the *Child Revised Impact of Events Scale* (CRIES‐8, 4‐point scale), a screening tool measuring symptoms of post‐trauma intrusion and avoidance, with good psychometric properties in war‐affected Arab populations (Punamäki, Palosaari, Diab, Peltonen, & Qouta, 2015; Veronese & Pepe, 2013); we relied on dimensional scores, noting that CRIES ≥17 points is predictive of symptoms of post‐traumatic stress disorder (http://www.childrenandwar.org).

In addition to education, displacement and demographic data, we assessed trauma exposure with the *Traumatic Events Checklist,* adapted from the Harvard Trauma Questionnaire and Gaza Checklist for use with adolescents in conflict settings (Panter‐Brick, Eggerman, Gonzalez, & Safdar, 2009). It features yes/no responses to 21 trauma items and an open‐ended section to identify any other traumas and contextualize experiences. We used a *Household Wealth Index* (ownership of 12 prespecified items) to demarcate wealth differentials in humanitarian settings (Panter‐Brick et al., 2009). We calculated the *dependency ratio* (dependents/nondependents) used for vulnerability assessment in Jordan (UNHCR Jordan, 2015). Finally, we recorded programme modality, session participation, sibling attendance, simultaneous engagement in concurrent humanitarian programmes and reasons for loss‐to‐follow‐up.

### Analyses

We confirmed instrument reliability in test–retest data and internal consistency with Cronbach's *α* (Panter‐Brick et al., 2017); all scales had acceptable *α* (HI *α* = .78, HD *α* = .83, PSS *α* = .74, AYMH *α* = .90, SDQ total difficulties *α* = .71, CRIES, *α* = .91), except for the SDQ prosocial (*α* = .55); psychometric data are shown in Table S1. There were no ceiling/floor effects in baseline scores (Terwee et al., 2007), except for SDQ prosocial (30.1% at the highest level of prosocial behaviour) and CRIES (37.7% with no symptoms of post‐traumatic stress reactions); such ceiling/floor effects were similarly distributed across intervention and control groups. We used all available data (item‐level missingness was <1%), without imputation. We compared baseline characteristics between treatment/control groups and cohorts retained/lost‐to‐follow‐up with independent sample *t*‐tests or chi‐square tests. We then examined individual‐level data for adolescents with complete measures at pre/postintervention.

For testing programme impacts, we ran multiple regression models on T2 endline scores, adjusted for T1 baseline symptom scores, to estimate the influences of study arm, age, gender, trauma exposure and programme implementation cycle. In additional models, we included interaction terms (age*study arm, gender*study arm, trauma exposure*study arm) and potentially moderating and mediating variables (household wealth, child/parental education status, time since displacement). To examine potential differential impacts, we used stratified regression models by lifetime trauma exposure (above/below the mean number of traumas for the cohort), and included programme modality to compare skills and activity‐based programme components. With T3 follow‐up data, we repeated analyses to examine outcome trajectories for youth in the treatment group, relative to controls, adjusting for programme implementation cycle, age, gender and other variables of interest. We calculated effects sizes with Cohen's *d*, a measure to convey the substantive significance of research findings.

We examined the consistency of findings across cycle‐specific and cycle‐pooled datasets (adjusting for cycle). In cycle 1 of data collection, a concern was the potential noncomparability between intervention and control groups, due to the nonrandomized selection criteria. We conducted a propensity score matched (PSM) sensitivity analysis (Garrido et al., 2014) on cycle 1 data, based on caliper of 0.25 nearest neighbour, with replacement, and the complete variable list used in regression models, to address potential noncomparability (selection bias) between intervention and control groups. We chose PSM rather than regression discontinuity design to improve comparability across groups, given that the latter requires determination of a priori, clinically meaningful cut‐offs for each outcome (Imbens & Lemieux, 2008). Having checked the robustness of regression models for cycle 1, and examined potential selection bias with respect to baseline characteristics of intervention and control groups for both cycles, we tabulated the comparable findings from regression models for both cycle‐specific and cycle‐pooled data.

Given the accessibility of concurrent interventions targeting youth affected by the Syrian crisis, we conducted analyses for the intent‐to‐treat sample (including all youth eligible for the study trial with pre/postintervention data in treatment and control groups) and for the *Advancing Adolescents* sample (excluding youth exposed to concurrent interventions and controls receiving treatment before the end of study). To ensure that observed effects were attributable to programme participation, the *Advancing Adolescents* sample thus excluded youth who engaged in other humanitarian programmes since baseline; to prospectively compare youth exposed versus not exposed to the intervention, it also excluded wait‐list controls who accessed the programme before follow‐up. We conducted primary analyses for the *Advancing Adolescents* sample, and sensitivity analyses with the intent‐to‐treat sample, at both T2 and T3 time‐points. We prospectively examined outcome trajectories for youth in the treatment group, relative to controls, adjusting for programme implementation cycle, age, gender and other variables. All analyses were performed with Stata 14.1, using svy commands to accurately estimate standard errors for data potentially clustered by family (siblings) and study site.

## Results

### Baseline characteristics

At baseline (Table S2), study trial participants included 817 adolescents (54.59% Syrian, 43.08% female), averaging 14.37 (*SD* 1.72) years of age, three‐quarters of whom (*n* = 603, 73.81%) were in cycle 2 randomized to treatment and wait‐list control groups. Participants in both implementation cycles had similar demographic characteristics, including trauma exposure and timeline since the most distressing trauma; as anticipated, however, those in cycle 1 (offered first‐priority access in humanitarian programming) averaged higher baseline scores for HD, AYMH, and SDQ difficulties (all *p* < .001). As expected, Syrian and Jordanian samples were similar in age, gender and treatment/control assignment, but differed in measures of wealth and wellbeing: Syrian refugees were poorer (Household Wealth Index, *p* < .001) and reported higher symptom scores for all stress/mental health outcomes (*p* = .016 to *p* < .001). With respect to gender, girls reported higher baseline symptoms for all outcomes (*p* < .003 to *p* < .0001) except prosocial behaviour and post‐traumatic stress reactions. Regional and international measures of distress and mental health difficulties were strongly correlated (e.g. HD and PSS, AYMH and SDQ difficulties, *p* < .0001). Fifty‐three per cent (*n* = 433) of participants experienced high insecurity levels, answering always/most of the time to items on the HI scale.

Lifetime trauma exposure averaged 3.95 (*SD* 3.73) events for the cohort, with 6.36 (*SD* 3.25) trauma events for Syrian refugees and 1.08 (*SD* 1.63) trauma events for Jordanians (*p* < .0001). Given that 82.5% of Syrian refugees reported ≥4 lifetime traumas, as compared to just 9.2% of Jordanians, categories of high/low‐trauma exposure overlap with refugee/nonrefugee status. For Syrians, the most often‐reported traumas were having witnessed bombardments (80.7%), having their homes forcibly searched by militia (71.5%), having seen homes demolished by militia (54.9%) and having seen wounded/dead bodies (53.8%). For Jordanians, these were having seen someone severely beaten (16.4%), had no access to medical care when severely ill (15.9%), and been in a bad accident (12.4%). Forty‐four per cent (*n* = 358) of study participants had baseline CRIES scores ≥17 points predictive of post‐traumatic stress reactions. Refugees had been displaced from Syria on average 2.78 (*SD* 0.95) years; in terms of dependency ratio, half (50.3%) were classed as severely vulnerable, corresponding with published data for Syrian refugees in Jordan (51.0%, dependency ratio ≥1.8; UNHCR Jordan, 2015).

### Treatment and control groups

Youth assigned to treatment and control groups were similar with respect to all baseline measures, for cycle‐specific and cycle‐pooled data, in the cohort retained to T2 endline and the cohort retained to T3 follow‐up (Table 2). At baseline, twenty per cent (*n* = 163) of participants had a sibling in treatment/control groups; however, all but 11 sibling pairs engaged in the same arm (regression models included siblings and adjusted for potential clustering). We included siblings on the recommendation of Mercy Corps, in order to respect the wishes of local families, so that girls could be accompanied to youth centres by their brothers.

**Table 2:** Sample characteristics at baseline for youth with pre/postintervention data, by treatment and wait‐list control groups

	*Advancing Adolescents* Cohort at postintervention (T2)				*Advancing Adolescents* Cohort at follow‐up (T3)			
	Overall	Control	Treatment		Overall	Control	Treatment	
	*n* = 463	*n* = 194 (41.9%)	*n* = 269 (58.1%)	*p* Value	*n* = 212	100 (47.2%)	112 (52.8%)	*p* Value
*Demographics*								
Gender, female *n* (%)	216 (46.7)	86 (44.3)	130 (48.3)	.40	90 (42.5)	45 (45.0)	45 (40.2)	.49
Nationality/refugee status, Syrian *n* (%)	250 (54.0)	113 (58.2)	137 (50.9)	.13	123 (58.0)	60 (60.0)	63 (56.3)	.68
Age (year)	14.37 (1.69)	14.22 (1.64)	14.48 (1.72)	.11	14.25 (1.73)	14.31 (1.69)	14.21 (1.77)	.66
Trauma (*N* lifetime events)	3.80 (3.66)	3.83 (3.67)	3.77 (3.66)	.86	4.09 (3.59)	4.37 (3.94)	3.85 (3.24)	.29
Education (highest grade, 0–12)	7.04 (2.15)	6.86 (2.21)	7.18 (2.10)	.58	6.98 (2.16)	6.9 (2.08)	7.05 (2.24)	.61
Household Wealth Index (*n* items)	7.88 (2.88)	7.97 (2.76)	7.81 (2.98)	.58	7.67 (2.99)	7.67 (2.76)	7.66 (3.19)	.98
Household dependency ratio	1.10 (1.22)	1.97 (1.63)	1.82 (1.56)	.11	1.01 (1.10)	0.99 (1.25)	1.02 (0.94)	.84
Displacement from Syria (year), Syrians only	2.78 (1.06)	2.84 (1.11)	2.73 (1.01)	.43	2.85 (1.21)	2.84 (1.25)	2.85 (1.18)	.96
*Primary outcomes*								
Human Insecurity (HI)	63.97 (21.20)	64.76 (21.36)	63.40 (21.11)	.50	65.49 (20.94)	65.47 (20.95)	65.51 (21.03)	.87
Human Distress (HD)	37.32 (20.52)	36.71 (19.50)	37.77 (21.26)	.59	36.24 (19.91)	37.47 (20.56)	35.14 (19.32)	.86
Perceived Stress (PSS)	27.49 (6.27)	27.69 (5.44)	27.36 (6.82)	.58	27.69 (6.24)	28.36 (5.65)	27.09 (6.70)	.24
*Secondary outcomes*								
Arab Youth Mental Health (AYMH)	34.10 (8.57)	34.18 (8.12)	34.04 (8.89)	.87	33.89 (8.16)	34.99 (8.46)	32.91 (7.78)	.47
SDQ total difficulties	14.93 (6.13)	14.85 (5.90)	14.99 (6.29)	.81	14.75 (5.77)	14.89 (5.73)	14.63 (5.84)	.94
SDQ prosocial	8.21 (1.74)	8.18 (1.83)	8.24 (1.68)	.68	8.32 (1.65)	8.44 (1.61)	8.21 (1.68)	.22
*Other outcomes*								
Post‐traumatic CRIES	12.98 (12.81)	12.11 (12.60)	13.59 (12.94)	.22	13.23 (12.23)	12.49 (12.29)	13.88 (2.20)	.89

*p* Value based on *t*‐tests on means and chi‐squares on frequencies; Data are Mean (*SD*), unless otherwise indicated as *n* (%).

### Postintervention cohort

Cohorts lost versus retained to T2 were similar with respect to all measures, except for education grade attained (*p* = .04; Table S2). Postintervention, sample attrition (*n* = 284) was 34.8% of baseline: youth were unreachable (*n* = 222 phone unanswered or disconnected), unavailable (*n* = 57 travelled away, busy with school/work, ill, married), or families declined to participate (*n* = 5). Of the 533 participants retained to T2, 70 youth (30 treatment, 40 control) engaged in multiple humanitarian programming, receiving more than the intended treatment, or receiving a treatment as controls; they were excluded from the *Advancing Adolescents* sample for primary analyses (*n* = 463). Youth engaged in multiple programmes were more likely to be younger Syrian boys (*p* = .03), reporting higher trauma exposure (*p* = .01). Locally, alternative services included: nonformal education and civic engagement programmes provided by Princess Basma Youth Resource Centre, the National Democratic Institute, Questscope and Save the Children; skills‐building programs provided by Injaz; training programmes provided by Princess Salma Center & Library for Information Technology; psychosocial support and recreational activities provided by International Medical Corps and UNICEF Jordan.

### Programme effectiveness

**Figure 2:**
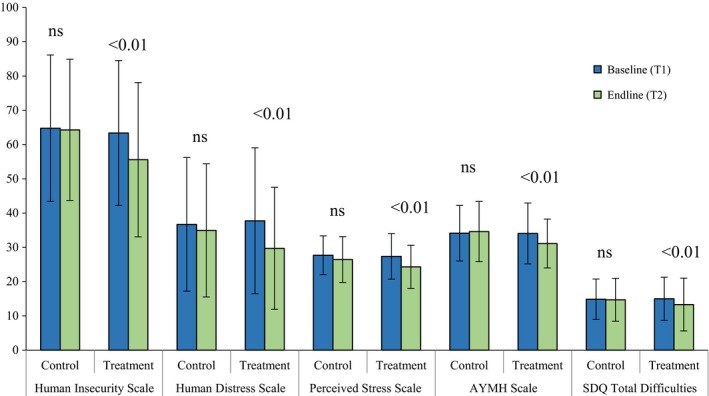
Primary health outcomes, pre/postintervention symptom scores (*n* = 463). Significance levels are for differences across time, within treatment and within control groups [Colour figure can be viewed at http://wileyonlinelibrary.com]

We examined the *Advancing Adolescents* sample with T1/T2 data (*n* = 463, 54.0% Syrian, 46.7% female). Session participation was high: 90% of intervention youth completed all 16 sessions offered. Univariate analyses regarding insecurity, stress and mental health difficulties – illustrated for cycle‐pooled data in Figure 2 – showed significant alleviation of symptom scores in the treatment group. This was largely driven by cycle 1 individuals who averaged relatively higher baseline symptom scores. There were no detectable treatment/control differences for prosocial behaviour and post‐traumatic CRIES.

We ran multiple regression models for cycle‐specific and cycle‐pooled data (Table 3). Results for cycle 1 demonstrated strong beneficial impacts of programme participation (*p* < .0001 for all outcomes except SDQ prosocial and CRIES scores), while those for cycle 2 showed significant impacts for AYMH (*p* < .01) only. Cycle 1 sensitivity analyses, with propensity score matching, confirmed the results of multiple regression modelling, in both beta coefficient magnitude and statistical significance (except for HD where *p* = .08), indicating minimal selection bias effect on presented results. In pooled‐data, beneficial impacts were confirmed for all primary and secondary outcomes (HI, *β* = −7.04, *p* < .0001; HD, *β* = −5.78, *p* < .0001; PSS, *β* = −1.92, *p* < .001; AYMH, *β* = −3.35, *p* < .0001; SDQ difficulties, *β* = −1.46, *p* < .01), except SDQ prosocial (*β* = .16, n.s.). Findings corresponded to medium to small effect sizes (Cohen's *d* = −0.4 for HI and AYMH; *d* = −0.3 for HD and PSS; *d* = −0.2 for SDQ difficulties).

**Table 3:** Programme impacts at postintervention (T2) for *Advancing Adolescents* youth versus wait‐list controls (*n* = 463)

Cohorts	Study cycle 1				Study cycle 2							
	Experimental trial				Randomized trial				Pooled cycles 1 and 2			
	*β*	95% CI	*p* Value	Cohen's *d*	*β*	95% CI	*p* Value	Cohen's *d*	*β*	95% CI	*p* Value	Cohen's *d*
*Primary outcomes (regression models 1–3)*												
Human Insecurity (HI)	−17.24	(−24.19, −10.29)	**<.0001**	−0.84	−4.22	(−8.65, 0.21)	.06	−0.20	−7.04	(−10.90, −3.17)	**<.0001**	−0.40
Human Distress (HD)	−18.00	(−25.10, −10.90)	**<.0001**	−1.02	−2.53	(−6.04, 0.97)	.16	−0.14	−5.78	(−9.02, −2.54)	**<.0001**	−0.28
Perceived Stress (PSS)	−5.61	(−7.39, −3.84)	**<.0001**	−1.11	−0.84	(−2.15, 0.48)	.21	−0.16	−1.92	(−3.05, −0.79)	**<.001**	−0.34
*Secondary outcomes (regression models 4–6)*												
Arab Youth Mental Health (AYMH)	−7.40	(−9.93, −4.88)	**<.0001**	−1.19	−2.19	(−3.71, −0.67)	**<.01**	−0.31	−3.35	(−4.68, −2.02)	**<.0001**	−0.44
SDQ total difficulties	−4.95	(−6.71, −3.19)	**<.0001**	−1.10	−0.38	(−1.44, 0.67)	.48	−0.09	−1.46	(−2.42, −0.50)	**<.01**	−0.23
SDQ prosocial	0.61	(−0.08, 1.29)	.08	0.65	−0.01	(−0.37, 0.34)	.94	−0.04	0.16	(−0.15, 0.47)	.32	0.09
*Other outcomes (regression model 7)*												
Post‐traumatic CRIES	−1.87	(−5.65, 1.91)	.33	−0.10	−0.56	(−3.05, 1.94)	.66	−0.03	−0.94	(−3.07, 1.19)	.39	−0.13

Regression models on endline (T2) score, adjusted for baseline (T1) value of outcome measure, age, gender, trauma, and cycle (where pooled data), and clustering by family and study site.

*β* indicates adjusted beta coefficient. Significant *p* value shown in bold.

We repeated analyses with the intent‐to‐treat sample (Table 4). Our findings were similar, except for AYMH impacts which lost significance (*p* = .06) in the intent‐to‐treat analysis for cycle 2. Tabulations of data show that findings were consistent in size, direction and significance across the analytical and intent‐to‐treat samples (Tables 3 and 4), in terms of beta coefficients, significance levels and effect sizes.

**Table 4:** Programme impacts at postintervention (T2) for Intent‐to‐Treat youth versus wait‐list controls (*n* = 533)

Cohorts	Study cycle 1				Study cycle 2							
	Experimental trial				Randomized trial				Pooled cycles 1 and 2			
	*β*	95% CI	*p* Value	Cohen's *d*	*β*	95% CI	*p* Value	Cohen's *d*	*β*	95% CI	*p* Value	Cohen's *d*
*Primary outcomes (regression models 1–3)*												
Human Insecurity (HI)	−16.77	(−23.65, −9.89)	**<.0001**	−0.83	−3.58	(−7.62, 0.47)	.08	−0.17	−6.06	(−9.65, −2.46)	**<.001**	−0.37
Human Distress (HD)	−17.83	(−24.94, −10.71)	**<.0001**	−1.04	−1.74	(−4.91, −1.43)	.28	−0.11	−4.68	(−7.66, −1.70)	**<.01**	−0.25
Perceived Stress (PSS)	−5.60	(−7.37, −3.82)	**<.0001**	−1.13	−0.42	(−1.65, 0.81)	.50	−0.09	−1.45	(−2.54, −0.36)	**<.01**	−0.26
*Secondary outcomes (regression models 4–6)*												
Arab Youth Mental Health (AYMH)	−7.46	(−9.97, −4.96)	**<.0001**	−1.21	−1.41	(−2.86, 0.05)	.06	−0.17	−2.53	(−3.82, −1.23)	**<.001**	−0.33
SDQ total difficulties	−4.91	(−6.67, −3.16)	**<.0001**	−1.11	−0.17	(−1.13, 0.78)	.72	−0.05	−1.15	(−2.04, −0.26)	**.01**	−0.19
SDQ prosocial	0.63	(−0.06, 1.31)	.07	0.66	−0.04	(−0.35, 0.27)	.81	−0.04	0.12	(−0.16, 0.40)	.39	−0.07
*Other outcomes (regression model 7)*												
Post‐traumatic CRIES	−1.55	(−5.39, 2.29)	.43	−0.10	−0.64	(−2.96, 1.67)	.59	−0.04	−0.95	(−2.97, 1.07)	.36	−0.23

Regression models on endline (T2) score, adjusted for baseline (T1) value of outcome measure, age, gender, trauma, and cycle (where pooled data), and clustering by family and study site.

*β* indicates adjusted beta coefficient. Significant *p* value shown in bold.

### Differential impacts

To address the question on differential impacts, we stratified the cohort by high/low‐trauma (*n* = 217 with ≥4 trauma, *n* = 246 with <4 trauma), which effectively differentiated Syrian refugees from Jordanian hosts. Table 5 presents results for cycle 2 and pooled‐cycle data (stratified results for cycle 1 could not be returned due to insufficient sample size). We observed a pattern of stronger impacts for youth with higher trauma exposures. Thus, in pooled data, the high‐trauma treatment group, relative to high‐trauma controls, demonstrated lower endline scores for HI (*β* = −7.25, *p* < .01), HD (*β* = −8.20, *p* < .01), PSS (*β* = −3.40, *p* < .001), and AYMH (*β* = −4.20, *p* < .001), with marginally significant changes for SDQ difficulties (*β* = −1.27, *p* = .06). At low‐trauma exposure, the treatment group, relative to low‐trauma controls, showed reduction on HI (*β* = −6.96, *p* = .01), AYMH (*β* = −2.64, *p* < .01) and SDQ difficulties (*β* = −1.66, *p* = .02) scores, with marginally significant change on HD scores (*β* = −3.87, *p* = .06). In terms of effect size, the beneficial impacts of the intervention were stronger in the high‐trauma versus low‐trauma cohort, for insecurity (Cohen's *d* = −0.46 vs. *d* = −0.39), distress (*d* = −0.41 vs. *d* = −0.20), PSS (*d* = −0.55 vs. *d* = −0.19) and AYMH (*d* = −0.54 vs. *d* = −0.40), but not for SDQ difficulties (*d* = −0.07 vs. *d* = −0.39). For primary outcomes (HI, HD, PSS), findings corresponded to medium effect sizes for high‐trauma youth, and small‐to‐medium effect sizes for low‐trauma youth. For secondary outcomes, there were medium effect sizes for AYMH for both high‐ and low‐trauma youth, medium effect sizes for SDQ difficulties for the low‐trauma youth, and no effects for SDQ prosocial scores.

**Table 5:** Programme impacts stratified by trauma exposure at postintervention (T2) for *Advancing Adolescents* youth versus wait‐list controls (*n*‐463)

Cohorts	Study cycle 2								Pooled cycles 1 and 2							
	Low‐trauma exposure cohort				High‐trauma exposure cohort				Low‐trauma exposure cohort				High‐trauma exposure cohort			
	*β*	95% CI	*p* Value	Cohen's *d*	*β*	95% CI	*p* Value	Cohen's *d*	*β*	95% CI	*p* Value	Cohen's *d*	*β*	95% CI	*p* Value	Cohen's *d*
*Primary outcomes (regression models 1–3)*																
Human Insecurity (HI)	−4.01	(−10.40, 2.38)	.22	−0.17	−4.54	(−10.8, 1.72)	.15	−0.25	−6.96	(−12.48, −1.44)	**.01**	−0.39	−7.25	(−12.60, −1.90)	**<.01**	−0.46
Human Distress (HD)	−0.68	(−5.33, 3.98)	.78	−0.04	−4.76	(−9.97, 0.44)	.07	−0.28	−3.87	(−7.94, 0.20)	.06	−0.20	−8.20	(−13.28, −3.12)	**<.01**	−0.41
Perceived Stress (PSS)	0.49	(−1.15, 2.14)	.55	0.00	−2.48	(−4.44, −0.53)	**.01**	−0.44	−0.66	(−2.12, 0.79)	.37	−0.19	−3.40	(−5.07, −1.74)	**<.001**	−0.55
*Secondary outcomes (regression models 4–6)*																
Arab Youth Mental Health (AYMH)	−1.81	(−3.76, 0.13)	.07	−0.30	−2.71	(−5.08, −0.33)	**.03**	−0.35	−2.64	(−4.37, −0.91)	**<.01**	−0.40	−4.20	(−6.26, −2.14)	**<.001**	−0.54
SDQ total difficulties	−0.50	(−2.05, 1.06)	.53	−0.17	−0.46	(−1.90, 0.97)	.52	0.00	−1.66	(−3.03, −0.28)	**.02**	−0.39	−1.27	(−2.62, 0.07)	.06	−0.07
SDQ prosocial	0.11	(−0.38, 0.60)	.65	0.04	−0.21	(−0.71, 0.29)	.40	−0.12	0.38	(−0.05, − 0.80)	.08	0.19	−0.12	(−0.56, 0.32)	.60	−0.02
*Other outcomes (regression model 7)*																
Post‐traumatic CRIES	−0.50	(−4.66, 3.65)	.81	−0.08	−0.44	(−3.42, 2.55)	.77	−0.04	−0.39	(−2.84, 2.06)	.75	−0.09	−1.43	(−5.05, 2.20)	.44	−0.28

Regression models on endline (T2) score, adjusted for baseline (T1) value of outcome measures, age, gender, cycle (if pooled) and clustering by study site. Cycle 1 results unavailable due to sample size restrictions. High‐trauma: four or more events; low‐trauma: three or fewer events. *β* indicates adjusted beta coefficient. Significant *p* value shown in bold.

We then ran extended models on programme modality (Table 6). All categories of programme modalities showed impacts, with strongest effects for sessions building technical and vocational skills relative to fitness activities or arts and crafts. Thus, youth who accessed sessions focused on technical skills (computer repairs, English language, first aid) showed significant reductions in endline HI, HD, PSS, AYMH and SDQ difficulties scores (*p* < .01 to *p* < .001), relative to wait‐list controls. Youth who accessed vocational skills (hairdressing, beautician training) showed significant reductions in HI, PSS and AYMH (*p* = .01 to *p* < .001), relative to controls. Youth who accessed programme sessions structured around fitness activities or arts and crafts showed improvements in one or two outcomes, particularly AYMH (*p* = .01 for fitness, *p* = .03 for arts and crafts), relative to controls, adjusting for baseline scores.

**Table 6:** Programme impacts by modality at postintervention (T2) for *Advancing Adolescents* youth versus wait‐list controls (*n* = 463)

Cohorts	Fitness			Arts and crafts			Vocational skills			Technical skills		
	*β*	95% CI	*p* Value	*β*	95% CI	*p* Value	*β*	95% CI	*p* Value	*β*	95% CI	*p* Value
*Primary outcomes (regression models 1–3)*												
Human Insecurity (HI)	−5.58	(−12.88, 1.73)	.13	−6.01	(13.19, 1.17)	.10	−7.92	(−13.46, −2.39)	**.01**	−7.14	(−12.05, −2.22)	**<.01**
Human Distress (HD)	−4.64	(−12.12, 2.83)	.22	−7.96	(13.76, −2.17)	**.01**	−3.68	(−7.87, 0.51)	.09	−7.52	(−11.07, −3.97)	**<.001**
Perceived Stress (PSS)	−0.93	(−12.12, 2.83)	.32	0.35	(−13.76, −2.17)	.70	−2.70	(−7.87, 0.51)	**<.01**	−2.54	(−11.07, −3.97)	**<.001**
*Secondary outcomes (regression models 4–6)*												
Arab Youth Mental Health (AYMH)	−2.92	(−5.23, −0.61)	**.01**	−2.29	(−4.32, −0.27)	**.03**	−3.30	(−5.05, −1.55)	**<.001**	−4.10	(−5.89, −2.31)	**<.001**
SDQ total difficulties	−2.17	(−3.94, −0.40)	**.02**	−1.19	(2.55, 0.18)	.09	−0.62	(−1.79, 0.55)	.30	−2.24	(−3.71, −0.77)	**<.01**
SDQ prosocial	0.24	(−0.32, 0.80)	.40	−0.28	(−0.80, 0.25)	.30	0.20	(−0.22, 0.61)	.35	0.30	(−0.10, 0.69)	.14
*Other outcomes (regression model 7)*												
Post‐traumatic CRIES	−0.60	(−4.40, 3.21)	.76	1.36	(−2.41, 5.13)	.48	−1.84	(−4.65, 0.96)	.20	−1.24	(−4.28, 1.79)	.42

Regression models on endline (T2) score, adjusted for baseline (T1) value of outcome measure, programme cycle, and clustering by family and study site. *β* indicates adjusted beta coefficient. Significant *p* value shown in bold.

In all models, we noted that trauma exposures, but not age or gender, were robust, independent predictors of endline symptom scores. We retained significant impacts when including a few significant interaction terms (AYMH, gender*treatment/control, *p* = .047; PSS, age*treatment/control, *p* = .02, and trauma*treatment/control, *p* = .01; data not shown).

### Sustained effects at follow‐up

Finally, we examined the cohort retained to follow‐up assessment: T3 corresponded on average to 11 months after the end of the intervention (falling 14 months after cycle 1 and 7 months after cycle 2). We retained 302 participants, with 43.5% sample attrition (*n* = 231) due to youth being unreachable (*n* = 135), unavailable (*n* = 77) or families declining to participate (*n* = 19). Participants retained to T3 were similar to those lost‐to‐follow‐up with respect to age, gender, and study arm allocation; most were cycle 2 participants (*n* = 250, 82.5%), and most were Syrian refugees (*n* = 184, 60.7%) who reported relatively less wealth (*p* = .001), higher trauma exposure (*p* = .003), and greater insecurity at baseline (*p* = .01) than youth lost‐to‐follow‐up (Table S2).

To answer the question of sustained impact, we compared youth receiving *Advancing Adolescents* (*n* = 112) with those still wait‐listed (*n* = 100) at T3: we excluded 90 youth who, as controls, had gained access to the intervention, or who, as treatment, had engaged in multiple humanitarian programming. These 90 youth were similar to youth retained for the analytical sample in all characteristics, except for higher trauma exposure (*p* = .04). This ensured that the *Advancing Adolescents* sample (*n* = 212) had comparable groups of youth receiving the intended treatment or no treatment. The corresponding intent‐to‐treat sample (*n* = 302) included all youth with follow‐up data.

Trajectories for cycle‐specific and pooled‐cycle data indicated that symptom scores alleviated for both treatment and control groups over time (Figure S1). The overall trend was towards recovery. Regression modelling for the *Advancing Adolescents* sample (Table 7) confirmed that, relative to controls, significant programme impacts persisted for human insecurity (*β* = −10.03, *p* < .001) and distress (*β* = −5.37, *p* = .03), adjusting for baseline symptom scores, age, gender, trauma exposure and programme implementation cycle. Effect sizes for insecurity (*d* = −0.42) and distress (*d* = −0.31) remained medium to small. Analyses for the intent‐to‐treat sample (*n* = 302) showed that youth randomized to the intervention group, as compared to youth assigned to the control group, showed sustained impacts for human insecurity (*β* = −6.09, *p* < .01) and a small effect size (*d* = −0.23).

**Table 7:** Programme impacts at follow‐up (T3) for *Advancing Adolescents* youth versus wait‐list controls (*n* = 212) and Intent‐to‐Treat youth versus wait‐list controls (*n* = 302)

	*Advancing Adolescents* sample at T3				Intent‐to‐Treat sample at T3			
	Pooled cycles 1 and 2				Pooled cycles 1 and 2			
	*β*	95% CI	*p* Value	Cohen's *d*	*β*	95% CI	*p* Value	Cohen's *d*
*Primary outcomes (regression models 1–3)*								
Human Insecurity (HI)	−10.03	(−15.18, −4.88)	**<.001**	−0.42	−6.09	(−10.59, −1.58)	**<.01**	−0.23
Human Distress (HD)	−5.37	(−10.16, −0.58)	**.03**	−0.31	−2.71	(−6.95, 1.53)	.21	−0.12
Perceived Stress (PSS)	−0.89	(−3.32, 1.53)	.47	−0.18	0.23	(−1.86, 2.32)	.83	−0.01
*Secondary outcomes (regression models 4–6)*								
Arab Youth Mental Health (AYMH)	−1.44	(−3.48, 0.60)	.17	−0.29	−0.80	(−2.70, 1.10)	.41	−0.12
SDQ total difficulties	−0.85	(−2.31, 0.60)	.25	−0.22	−0.50	(−1.79, 0.78)	.44	−0.11
SDQ prosocial	−0.38	(−0.81, 0.05)	.08	−0.25	−0.22	(−0.58, 0.15)	.24	−0.18
*Other outcomes (regression model 7)*								
Post‐traumatic CRIES	−0.86	(−4.434, −2.71)	.63	−0.02	0.93	(−2.14, 4.00)	.55	0.14

Regression models on follow‐up (T3) score, adjusted for baseline (T1) value of outcome measure, programme cycle, and clustering by family and study site.

*β* indicates adjusted beta coefficient. Significant *p* value shown in bold.

## Discussion

We studied the outcomes of a structured programme initiated to alleviate profound stress and enhance emotional and behavioural regulation in war‐affected adolescents. The programme was a brief, scalable and replicable psychosocial intervention targeting a potential ‘lost generation’ impacted by the Syria crisis (Kurtz, 2016). It modelled several elements thought to be key features of successful MHPSS interventions: it offered a group‐based programme, with emphasis on socially meaningful activities, delivered by well‐trained providers, following specific mobilization and community engagement (Bangpan et al., 2017). Our findings indicate that the intervention led to improved psychosocial wellbeing for Syrian refugee and Jordanian host‐community 12–18 year‐old adolescents eligible for humanitarian support, both girls and boys. Half our baseline sample (53.0%, *n* = 817) reported high insecurity levels, responding “always” or “most of the time” to items on the Human Insecurity Scale. This compares to 63.9% of Palestinians in a study (*n* = 1,778) of the West Bank, East Jerusalem and Gaza (Barber et al., 2016). Relative to controls in pooled‐cycle data, adolescents who completed the 8‐week programme decreased Human Insecurity scores by 7.04 percentage points (CI −10.90, −3.17), with similarly substantial decreases on Human Distress and AYMH scores (Table 3). The HI, HD and AYMH are three screening tools designed for face validity, sensitivity and relevance to the region. The SDQ, however, has clinical validity: each 1‐point increase in child‐reported mental health difficulties increases the probability of clinician‐assigned mental disorder (Goodman & Goodman, 2009). Programme beneficiaries averaged SDQ difficulty scores that, while similar at baseline, were 1.46 points (CI −2.44, −0.49) lower than controls in models adjusted for trauma, age, gender and programme implementation cycle.

We looked to effect sizes to help evaluate the overarching question of programme effectiveness (how well does an intervention work, in a range of contexts?). Given our effect size (*d* = −0.4) for human insecurity, the average youth in the treatment group was 0.4 standard deviations below the average youth in the control group, thus showing fewer symptoms of insecurity than 66% of controls. For human distress (*d* = −0.3), the average youth completing the intervention had a lower score than 62% of controls. These effects sizes thus suggest intervention impacts of substantive relevance in promoting wellbeing. Further, effect sizes were greater for youth exposed to 4 or more traumas, for all outcomes other than SDQ (Table 5).

Overall, our results suggest medium to small effect sizes (−0.4 for HI and AYMH; −0.3 for HD and PSS; −0.2 for SDQ difficulties), equivalent or greater to observed impacts for comparable skills and activity‐based interventions delivered to this age group in crisis settings (Jordans et al., 2009; Metzler & Ager, 2015; Tol et al., 2011). A global review of ‘Child Friendly Spaces’ (which engage youth in group‐based activities, including life skills, sports, arts/folklore and vocational training) reported a wide range of impacts on psychosocial wellbeing (Ager, Metzler, Vojta, & Savage, 2013) and weighted average effect sizes of −0.2 on outcomes such as AYMH and SDQ (Metzler, Savage, Yamano, & Ager, 2015). Small effect sizes were also reported for two‐generation approaches delivering parenting and family skill training programs to displaced families (Annan, Sim, Puffer, Salhi, & Betancourt, 2016). In the context of a randomized control trial assessing psychotherapy‐based and activity‐based interventions, significant improvements for depression severity were observed for group interpersonal psychotherapy but not for creative play; there were no effects for other outcomes assessing anxiety, conduct and function scores (Bolton et al., 2007). In their systematic review, Bangpan et al. (2017) were able to compare clinical and psychosocial interventions for young people in humanitarian crises: while good‐quality evaluations of trauma‐focused, cognitive behavioural therapy (*n* = 3) yield effect sizes averaging −2.21 for PTSD, −1.76 for emotional problems, and −1.20 for conduct problems, good‐quality evaluations of psychosocial interventions (*n* ≤ 4) yield average effect sizes of −0.67 for PTSD, −0.98 for emotional problems and −0.45 for conduct problems (Bangpan et al., 2017).

The literature thus reports varying programme impacts, reflecting intervention goals (Bolton et al., 2007), features of programmatic content, delivery and quality (Bangpan et al., 2017; Brown, de Graaff, Annan, & Betancourt, 2017), characteristics of targeted beneficiaries such as gender, trauma exposure, or social support, and differences in contextual factors such as levels of human insecurity (Ziadni et al., 2011). This underscores the value of identifying common practice elements of interventions that are ‘active ingredients’ for targeted change (Brown et al., 2017). It also underscores the importance of reporting differential effects for subgroups of participants (Das et al., 2016) with respect to gender, age, socioeconomic status and geographic settings, or as done in this study, with respect to trauma exposure as well as gender, age and cycle implementation.

Our results differed by programme implementation cycle: while both cycles demonstrate evidence of programme effect, impacts were muted in cycle 2. Such findings might be due to at least three variables: study design, participant characteristics and/or implementation factors. First, in terms of study design, guaranteed funding allowed a randomized controlled design to be ethically implemented in cycle 2, but not cycle 1. Other studies of war‐affected youth have also reported different results when evaluating the same behavioural intervention with open trials (Newnham et al., 2015) and randomized controlled trials (Betancourt et al., 2014). We conducted sensitivity analyses to reduce potential bias, improving comparability between treatment and controls (Durlak, 2008); these confirmed cycle 1 results. Second, with respect to participant characteristics, youth were prioritized for program access in terms of vulnerability and need, which Mercy Corps staff established on the basis of children's reported mental health difficulties and access to services. Relative to cycle 2, cycle 1 participants showed higher baseline scores (*p* < .001) for Human Distress, AYMH, and SDQ difficulties, but similar trauma exposure and time since trauma. Having explicitly adjusted for baseline scores and programme cycle in pooled‐data regression models, we believe that differential impact across cycles was driven by the higher baseline symptoms documented in cycle 1. Third, with respect to implementation, adaptation in the delivery of programme modalities and availability of alternative services are important – but often overlooked – factors potentially influencing effect sizes. We noted that Mercy Corps began with offering a larger number of programme modalities to enhance programme diffusion in cycle 1, and that alternative humanitarian services were more readily accessed by youth in cycle 2, reflecting increased donor funding in the humanitarian area.

Our primary analyses were based on an analytical cohort comparing treatment versus control groups, excluding youth accessing alternative humanitarian services and wait‐listed youth receiving *Advancing Adolescents* before the end of study. As Durlak (1985) once emphasized: “Monitoring of comparison groups is important. It is often assumed incorrectly that controls do not receive any services, but this is almost never the case […] Child psychotherapy studies in which alternate conditions have received treatment have yielded mean effect sizes half as large in magnitude as those produced in true treatment versus control designs” (p. 329). In our study, we were able to monitor whether youth were exclusively or nonexclusively engaged in the *Advancing Adolescents* programme, and compare results based on the analytical and intent‐to‐treat samples. At 10 weeks postintervention (T2), we observed similar findings in size, direction and significance across the two samples (Tables 3 and 4), while at 11‐month follow‐up (T3), the sustained effects for the analytical cohort were attenuated in the intent‐to‐treat sample (Table 7). In our view, it is especially important for program evaluations to monitor the receipt of alternative services in areas of protracted conflict receiving an influx of humanitarian interventions. In Durlak's review of why implementation matters for assessing programme outcomes (p. 329), the monitoring of comparison groups helps to provide “a more accurate view of the value of a new intervention.” Careful attention to questions of implementation science is increasingly required in mental health program evaluations in low‐resource or conflict settings (Brown et al., 2017; Murray et al., 2014).

Importantly, findings indicate that adolescents with higher trauma exposure benefited most from programme participation, as shown by analyses for cohorts stratified by lifetime trauma (Table 4). Our threshold of trauma exposure (four or more events) reflects a population‐specific experience of childhood adversity among Syrian refugees and host‐community youth. As other studies have shown, experiencing this number of adverse childhood experiences has long‐term consequences for human health and development (Felliti, 2002). Thus in WHO mental health surveys, clusters of childhood adversities predict not just the occurrence but also the onset of significant psychopathology in later adulthood (Kessler et al., 2010). Recent reviews of the biological consequences of violence also emphasize how toxic stress and multiple trauma experienced in childhood have likely cognitive, physiological and epigenetic consequences across generations (Leckman, Panter‐Brick, & Saleh, 2014). Psychosocial support for those who experience multiple childhood adversities is thus a key priority for humanitarian policy and practice.

In contrast to measures of insecurity, distress and mental health difficulties, we found no discernable impacts for measures of post‐traumatic stress reactions and prosocial behaviour, as compared to controls. Previous meta‐analyses of MHPSS interventions for children exposed to trauma have shown heterogeneous results, especially for PTSD (Tol et al., 2011). Additional programming responses are required to address PTSD impairment within a coherent system of interventions for mental health and psychosocial support (Hassan et al., 2016; Quosh et al., 2013). In turn, the lack of change in self‐report SDQ prosocial scores may have been due to ceiling effects in baseline scores observed in this study, or to insufficient reliability of this subscale as noted elsewhere (Muris, Meesters, & van den Berg, 2003). Changes in prosocial behaviour may also require more than brief interventions using group‐based structured activities, such as sustained support at family and community level.

Although we found that the *Advancing Adolescents* programme has no discernable effects on prosocial behaviour, Mercy Corps' in‐house evaluation did report positive impacts in terms of social networks and trust, perception of safety in their community, and confidence in the future (Kurtz, 2016). Specifically, participants reported an increase in the number of friends outside of their own community, and built trust within a diverse group of peers through programme activities that brought together small groups of young people in a safe environment to share ideas and accomplishments. These results were important “in light of the isolation and social tension affecting young people in Jordan, [given that] building connections and trust between Syrian and Jordanians is critical to reducing the tensions and the likelihood of violence between the groups” (Kurtz, 2016, 5). In focus group discussions with the families of participants, one father stated: “When we first arrived to Jordan, people would call me the Syrian. Now people call me Abu Mohammed and pay my house a visit,” and another said: “my son has learned to accept others and avoid judgment” (Mercy Corps, 2016). Strengthening elements of social cohesion and social stability, however, is not necessarily the same as strengthening youth prosocial behaviour.

We found no consistent influence of gender and age across our broad range of outcomes, for programme sessions conducted in gender‐specific and, where possible, age‐differentiated groups. We did find evidence for stronger impacts for sessions that imparted technical and vocational skills, relative to fitness, arts and crafts (Table 6). One reason for this may have been that sessions based on technical and vocational skills were viewed as having potential socioeconomic benefit: in focus group discussions, families expressed strong preferences for training sessions that helped young people learn transferable skills (Mercy Corps, 2016). Accordingly, future evaluations might clarify whether offering a range of modalities constitutes a ‘surface’ adaptation, or is a ‘pivotal’ component of programme implementation (Durlak, 2008). This is especially important where humanitarian organizations adapt their programmes to improve sociocultural relevance and to reflect changing mandates. In Jordan for example, Mercy Corps recently expanded *Advancing Adolescents* to include parents in stress attunement training, and also incorporated this theoretical lens and structured, group‐based activities into humanitarian programming to counter violent extremism.

Finally, the follow‐up (T3) data indicated that, at cohort‐level, youth wellbeing improved over time, even in the absence of humanitarian programming. Specifically, we found sustained intervention effects for human insecurity (Table 7); for other outcomes, symptom alleviation occurred in both the intervention and the control groups. It is likely that while individual‐level interventions work to accelerate processes of recovery and adjustment, additional family‐level and community‐level support are needed to secure longer term benefits (Ager & Metzler, 2017). Reducing ‘latency to recovery’ is important in meeting the humanitarian imperative to improve lives, alleviate suffering, and by extension, enable vulnerable youth to take up potential opportunities for educational and social advancement. However, the evaluation of sustained effects will require more frequent follow‐up assessments. Small to moderate intervention impacts will likely diminish over time, especially in the absence of sustained programming aimed at improving family‐level circumstances.

The study had four main limitations. First, we evaluated two out of five cycles of programme implementation. For ethical reasons, we enlisted wait‐listed participants as controls in cycle 1, randomized assignment to wait‐listed controls in cycle 2, and analytically adjusted for study design. Propensity score matched sensitivity analyses for cycle 1 findings confirmed that results of multiple regression analyses were not biased by the potential noncomparability of intervention and control participants due to nonrandomized group assignment. Second, we did not assess variation in the quality of programme delivery, potentially an important predictor of impact (Metzler et al., 2015), and were only able to account for potential contamination across treatment and control groups through analytical strategy. Third, while we paid careful attention to variables such as wealth, education and displacement history, we did not measure family‐level variables, such as parent support, likely to affect adolescent mental health trajectories (Betancourt & Khan, 2008; Betancourt, McBain, Newnham, & Brennan, 2013a; Jordans et al., 2009; Murphy, Rodrigues, Costigan, & Annan, 2017; Panter‐Brick et al., 2009; Panter‐Brick, Grimon, & Eggerman, 2014). Finally, current analyses are based on self‐reports; however, time‐series analyses of scalp hair cortisol, a physiological biomarker of chronic stress levels, corroborate self‐reported data on intervention impacts (Dajani, Hadfield, van Uum, Greff, & Panter‐Brick, under review).

## Conclusions

There is a dearth of high‐quality, multioutcome research evaluating mental health and psychosocial interventions for children and adolescents in conflict settings, particularly in Middle Eastern contexts. The poor evidence base leads to a lack of credibility with respect to mental health treatment and prevention (Patel, 2014). We show that a skills and activity‐based psychosocial intervention had positive impacts on adolescent wellbeing, a finding of relevance to humanitarian, scientific and policy concerns in resource‐constrained settings. Thus *Advancing Adolescents* programme had beneficial impacts both on symptoms of insecurity and distress and on emotional and behavioural difficulties. We also show differential impacts on outcomes capturing human insecurity, distress and mental health, for youth with different levels of trauma exposure and childhood adversities. This has important implications for the design of humanitarian interventions seeking to improve child and adolescent developmental and health outcomes, and underscores the importance of providing scalable interventions to protect war‐affected youth, especially those with multiple trauma exposures.

As discussed above, future health‐related programme evaluation research conducted in conflict‐affected settings will need to carefully document on‐the‐ground implementation, differential programme impacts, pivotal programme components and latency to recovery. Indeed, the field of dissemination and implementation science is one where it is vital to address questions of ‘process’ regarding how evidence‐based interventions are put into practice (Brown et al., 2017; Murray et al., 2014). We found, for example, that local leadership was essential to the conduct of a randomized control trial: families consented to a random allocation to treatment and wait‐list controls because the ultimate fairness of this study design was explained and endorsed by a Jordanian/Syrian scientist (RD), well‐known and trusted in the area. Moreover, our program evaluation was initiated at the request of Mercy Corps, which meant that the study trial benefited from close collaboration between programme implementation and programme evaluation teams. The study trial was, however, financed and staffed independently from Mercy Corps; it came under the umbrella of a funding initiative that enabled partnerships between research and humanitarian organizations. Issues of trust, partnership, leadership and structured funding are among those that matter most for ensuring research and implementation in conflict settings.

The alleviation of distress, insecurity and mental health difficulties, through a scalable, technically feasible programme of structured group‐based activities, helps fulfil the humanitarian mandate to reduce suffering. By incorporating features directed to stress attunement, it also informs several scientific goals: to build the evidence base for public health impacts in crisis settings (Blanchet et al., 2015), to alleviate the developmental and intergenerational consequences of toxic stress (Garner & Shonkoff, 2012; Leckman et al., 2014; Shonkoff et al., 2012), and to mitigate the long‐term clinical impacts of childhood adversities (Kessler et al., 2010). Psychosocial interventions have the potential to not only alleviate distress and insecurity, reducing the latency for recovery, but also to protect the developmental trajectories of youth facing the extreme disruption of protracted conflict and displacement. To achieve long‐term benefits, however, interventions will need to enhance familial and structural support, as well as the individual and interpersonal support provided in current programming.

**Key points**

- Previous reviews have demonstrated the need to strengthen the evidence base on mental health and psychosocial interventions for children and adolescents in areas affected by armed conflict.
- In response to the Syria crisis, initiatives to prevent the loss of an entire generation to the harmful effects of war and forced displacement have been implemented. We evaluated a structured, 8‐week psychosocial programme informed by a profound stress attunement framework targeted at refugee and host community adolescents.
- The intervention improved insecurity, distress and mental health difficulties, but not prosocial behaviour and post‐traumatic stress reactions. Beneficial impacts were strongest for adolescents who had experienced four or more lifetime traumas.
- Youth in both treatment and control groups tended to improve over time. However, with respect to levels of insecurity, those engaged in the psychosocial intervention had sustained benefits relative to youth who did not.
- Robust evidence of health interventions can inform strategies to offset the long‐term impacts of childhood adversities and protect the developmental consequences of youth facing the extreme disruption of protracted conflict and forced displacement.
- Future research in conflict‐affected settings needs to carefully document programme implementation, differential intervention impacts and latency to recovery. It best achieves this through building strong partnerships.

## Supporting information


**Figure S1.** Symptom scores at three time‐points for cycle‐specific and pooled‐cycle data for Advancing Adolescents. Significance levels are for differences across time, within treatment and within control groups.

**Table S1.** Correlations and internal consistency for baseline study variables and 7‐day test–retest reliability with a separate sample.
**Table S2.** Baseline (T1) characteristics of youth lost versus retained to study.

**Appendix S1.** CONSORT Checklist.
