# Supplementary material for: Insecurity, distress and mental health: experimental and randomized controlled trials of a psychosocial intervention for youth affected by the Syrian crisis
Source: J Child Psychol Psychiatry. 2017 Oct 2;59(5):523–41. doi: 10.1111/jcpp.12832 (PMC5972454; doi:10.1111/jcpp.12832)
Supplement: Supplementary file 1 — Figure S1. Symptom scores at three time‐points for cycle‐specific and pooled‐cycle data for Advancing Adolescents. Significance levels are for differences across time, within treatment and within control groups. [file JCPP-59-523-s001.docx]

Online Supporting Information for: Insecurity, distress, and mental health: Experimental and randomized controlled trials of a psychosocial intervention for youth affected by the Syrian Crisis – Catherine Panter-Brick et al.

**Online Figure S1.** Symptom scores at three time-points for cycle-specific and pooled-cycle data for Advancing Adolescents. Significance levels are for differences across time, within treatment and within control groups.

a. Cycle 1, experimental trial (retained to follow-up, n=36).

b. Cycle 2, randomized controlled trial (retained to follow-up, n=176).

c. Pooled-cycle data (retained to follow-up, n=212).
