# Supplementary material for: Insecurity, distress and mental health: experimental and randomized controlled trials of a psychosocial intervention for youth affected by the Syrian crisis
Source: J Child Psychol Psychiatry. 2017 Oct 2;59(5):523–41. doi: 10.1111/jcpp.12832 (PMC5972454; doi:10.1111/jcpp.12832)
Supplement: Supplementary file 2 — Table S1. Correlations and internal consistency for baseline study variables and 7‐day test–retest reliability with a separate sample. Table S2. Baseline (T1) characteristics of youth lost versus retained to study. [file JCPP-59-523-s002.docx]

Online Supporting Information for: Insecurity, distress, and mental health: Experimental and randomized controlled trials of a psychosocial intervention for youth affected by the Syrian Crisis – Catherine Panter-Brick et al.

**Online Table S1.** Correlations and internal consistency for baseline study variables and 7-day test-retest reliability with a separate sample.

|  | **Human Insecurity** | **Human Distress** | **Perceived Stress** | **Arab Youth Mental Health** | **SDQ total difficulties** | **SDQ prosocial** | **Child Revised Impact of Events** |
| --- | --- | --- | --- | --- | --- | --- | --- |
| **Pearson correlations (*n* = 817)** |  |  |  |  |  |  |  |
| Human Distress (HD) | .28** | - |  |  |  |  |  |
| Perceived Stress (PSS) | .19** | .49** | - |  |  |  |  |
| Arab Youth Mental Health (AYMH) | .23** | .59** | .51** | - |  |  |  |
| SDQ total difficulties | .15** | .49** | .50** | .57** | - |  |  |
| SDQ prosocial | .13** | -.09* | -.16** | -.07* | -.27** | - |  |
| Child Revised Impact of Events (CRIES) | .16** | .32** | .28** | .37** | .24** | .02 | - |
| **Chronbach’s alpha (*n* = 817)** |  |  |  |  |  |  |  |
|  | .78 | .83 | .74 | .90 | .71 | .55 | .91 |
| **Test-retest correlations (*n* = 41)** |  |  |  |  |  |  |  |
|  | .69** | .63** | .76** | .82** | .73** | .76** | .79** |

** *p* < .001, * *p* < .05, SDQ: Strengths and Difficulties

| **Online Table S2.** Baseline (T1) characteristics of youth lost vs. retained to study. | | | |  |  | | |  |  |  |
| --- | --- | --- | --- | --- | --- | --- | --- | --- | --- | --- |
|  |  |  |  |  | |  |  | | |  |
|  | **T1** | **T2** | | | | **T3** | | | | |
| **Cohort** | **Overall** | **Lost** | **Retained** | **Difference** | | **Lost** | **Retained** | | | **Difference** |
|  | **n=817** | **n=284** | **n=533** | **P level** | | **n=231** | **n=302** | | | **P level** |
| **Demographics** |  |  |  |  | |  |  | | |  |
| Gender, n (%) |  |  |  |  | |  |  | | |  |
| Male | 465 (56.92) | 171 (60.20) | 294 (55.09) | 0.15 | | 125 (54.60) | 169 (56.00) | | | 0.86 |
| Female | 352 (43.08) | 113 (39.80) | 239 (44.91) |  | | 106 (45.40) | 133 (44.00) | | |  |
| Nationality/refugee status, n (%) |  |  |  |  | |  |  | | |  |
| Jordanian | 371 (45.41) | 135 (47.50) | 236 (44.30) | 0.33 | | 117 (50.80) | 119 (39.40) | | | **<0.01** |
| Syrian | 446 (54.59) | 149 (52.50) | 297 (55.70) |  | | 114 (49.20) | 183 (60.60) | | |  |
| Study arm, n (%) |  |  |  |  | |  |  | | |  |
| Controls | 354 (43.33) | 120 (42.30) | 234 (43.90) | 0.71 | | 94 (40.30) | 140 (46.40) | | | 0.16 |
| Treatment | 463 (56.67) | 164 (57.70) | 299 (56.10) |  | | 137 (59.70) | 162 (53.60) | | |  |
| Program cycle, n (%) |  |  |  |  | |  |  | | |  |
| Cycle 1 | 214 (26.19) | 77 (27.02) | 137 (25.75) | 0.70 | | 84 (36.60) | 53 (17.50) | | | **<0.001** |
| Cycle 2 | 603 (73.81) | 207 (72.98) | 396 (74.25) | 0.68 | | 147 (63.40) | 249 (82.50) | | |  |
|  |  |  |  |  | |  |  | | |  |
| Age (yr) | 14.37 (1.72) | 14.51 (1.75) | 14.30 (1.70) | 0.10 | | 14.4 (1.62) | 14.21 (1.74) | | | 0.22 |
| Trauma (n lifetime events) | 3.95 (3.73) | 3.99 (3.81) | 3.95 (3.68) | 0.86 | | 3.41 (3.69) | 4.35 (3.65) | | | **<0.01** |
| Education (highest grade, 0-12) | 7.10 (2.15) | 7.33 (2.15) | 6.98 (2.14) | **0.04** | | 7.15 (2.04) | 6.86 (2.02) | | | 0.16 |
| Household Wealth Index (n items) | 7.96 (2.85) | 8.14 (2.81) | 7.87 (2.86) | 0.19 | | 8.39 (2.58) | 7.52 (2.98) | | | **<0.01** |
| Household Dependency Ratio | 1.10 (1.27) | 1.12 (1.36) | 1.08 (1.22) | 0.64 | | 1.07 (1.01) | 1.08 (1.29) | | | 0.82 |
| Displacement from Syria (yr), Syrians only | 2.78 (0.95) | 2.79 (0.78) | 2.78 (1.02) | 0.91 | | 2.72 (0.87) | 2.81 (1.08) | | | 0.45 |
| **Primary outcomes** |  |  |  |  | |  |  | | |  |
| Human Insecurity (HI) | 64.41 (21.29) | 63.80 (21.95) | 64.74 (20.95) | 0.53 | | 62.14 (21.27) | 66.88 (20.71) | | | **0.01** |
| Human Distress (HD) | 37.78 (20.83) | 38.07 (20.85) | 37.63 (20.85) | 0.81 | | 37.49 (20.51) | 37.75 (21.25) | | | 0.88 |
| Perceived Stress (PSS) | 27.46 (6.23) | 27.73 (6.28) | 27.32 (6.21) | 0.37 | | 27.17 (6.29) | 27.45 (6.11) | | | 0.58 |
| **Secondary outcomes** |  |  |  |  | |  |  | | |  |
| Arab Youth Mental Health (AYMH) | 34.09 (8.56) | 34.01 (8.28) | 34.13 (8.71) | 0.82 | | 33.86 (8.82 | 34.47 (8.79) | | | 0.51 |
| SDQ total difficulties | 14.87 (6.17) | 14.92 (6.25) | 14.84 (6.14) | 0.94 | | 14.75 (6.45) | 14.96 (5.88) | | | 0.72 |
| SDQ prosocial | 8.19 (1.74) | 8.12 (1.75) | 8.22 (1.73) | 0.48 | | 8.12 (1.79) | 8.3 (1.67) | | | 0.21 |
| **Other outcomes** |  |  |  |  | |  |  | | |  |
| Posttraumatic CRIES | 13.10 (12.67) | 3.01 (12.52) | 13.21 (12.76) | 0.76 | | 11.9 (12.94) | 14.28 (12.62) | | | 0.22 |
| P level based on t-tests on means and chi-squares on frequencies. Data are Means (SD), unless otherwise indicated as n (%) | | | | | | | | | |  |
